# The State’s Duty in Caring for Its Consumptives

**Published:** 1902-11

**Authors:** J. L. Hiers

**Affiliations:** Savannah, Ga.


### THE STATE'S DUTY IN CARING FOR ITS CONSUMPTIVES.*

By J. L. HIERS, M.D.,

SAVANNAH, GA.

As has often been declared, "the proper study of mankind is man." Personifying the State, as the guardian of the material welfare and prosperity of her people, it might be added with propriety that, for the maintenance of her honor and the edification of her subjects, the onward march of civilization demands that she shall follow where science, truth and health may direct.

Thus, in its final analysis, the State becomes in reality, and naturally, the custodian of the health of its citizens; that is, in so far as preventive medicine, its legitimate work, is concerned. So plain is its duty, so comprehensive its power for good, that scarcely a question arises, whether in the legislative halls, in politics, or religion, that has not a possible medical aspect, or on which scientific medicine cannot shed a ray of light. The penalty paid for lack of knowledge along lines conducive to good is beyond conception; but frequently it is great and alarming in the magnitude of its horror. "Public medicine in this wider sense in its relations to economics, legislation, and the like, is a field that is well worth cultivation." Unfortunately, at present those who make our laws apparently little appreciate the importance of enactments calculated to protect the consumptive or to promote his physical prosperity—to say nothing of the danger to the community from the too free license accorded him; and hence it devolves on us, assembled here with one purpose, to do what we can for their enlightenment.

So universal has tuberculosis become, so potent is its power for evil, so tenacious its hold on humanity, that measures have been adopted by different nations for its prevention. The movement was first inaugurated by the English, in 1836, when a law was passed providing for the construction of healthy houses. Since

*Read before the Georgia Sociological Society, Atlanta, June, 1902.

then more than ten additional acts of parliament to render salubrious the dwellings of the poor have been authorized, and in consequence the mortality from this disease has been reduced forty per cent. In 1889 the "National Association for the Prevention of Tuberculosis" was founded, and the present King Edward, then Prince of Wales, was chosen president. Its object was further prevention by educating the masses, and the good accomplished cannot be estimated. Germany founded societies for the construction of sanatoria, then similar organizations for the propagation of the idea, and published popular pamphlets for distribution among her people. Belgium has a National League against tuberculosis; and in Norway a princely sum was voted for the purpose of printing and distributing a work on the subject. The most eminent authorities in France have addressed meetings and explained the rules of prophylaxis. Thus gradually, in all countries, the public is beginning to realize that personal care and cleanliness are important factors, and that a consumptive is only dangerous when necessary precautions are belittled, condemned or neglected. The "old world" has become imbued with the spirit of the times; and notwithstanding that the freedom of a free people will not tolerate the imperiousness of a monarchy, we, to a degree at least, are striving to check the progress of the great white plague. Several States have taken steps already for its arrest; and the Government has established in New Mexico sanatoria for its infected sailors and soldiers. The success achieved has been so encouraging that we are justified in exulting; and the day can not be far distant when each State will recognize its responsibility in this respect and unite for the purpose of diminishing the prevalence and mortality of a scourge which is now "disabling to a greater or less extent a million and a quarter of the people of this country, and which kills four and a half times as many as smallpox, scarlet fever, typhoid fever and diphtheria combined."

If diagnosed in its incipiency and an effort be made to control it, tuberculosis, in the light of modern investigation, cannot be regarded with the gravity it once was. Pure, fresh air has done much to silence the alarm that formerly was so widely felt. Instead of being considered hereditary, incurable and ineradicable, it is now looked upon as acquired, to a great extent amenable to treatment, and possible of eradication. Having diagnosticated the disease, the responsibility of the physician becomes appreciably less. That is, his moral obligation to the community ceases when the people have been apprised of the danger that threatens and counseled as to the best method of escape. Generally speaking, doctors are not weighted with wealth, and if they detect the malady early, then the public at large, through the instrumentality of its opulent citizens or its municipal, State, or governmental authorities, is bound morally, economically and socially to afford the patient a chance for his life. As one author has tersely remarked: "It must not be care for the doomed and the dying—the reach must be greater and extend out to the home of the sufferer." Palatial edifices in overcrowded cities are monuments to the architect's skill, it is true; but misapplied philanthropy only serves to make more comfortable the untimely end of a life that might be otherwise rescued from its doom. Stem the tide from the country to the city's busy strife, turn it back in its course; let the influx be to the woods that are green and fields that are fair, where nature romps and gambols in a purer atmosphere and the air is free from noxious organisms and vaulting vampires, and soon the blush will return to their hectic faces, the luster will come back to their drooping eyes, hope will be sweeter to their heavy hearts, and they will be spared to bless humanity, their country and their God.

When we pause to wonder what this nation has done for the social and political upbuilding of man, we cannot but notice in marked contrast the almost puritanical conservatism displayed toward him when compassed about with sickness. Within the sacred circle of his home how often is he made to combat the ravages of disease, which, in many instances are incited through the rapacity of avaricious proprietors of unsanitary hovels, where the lower strata of the human family ekes out a miserable existence. But, on the other hand, fancy a wide-spread and fatal epidemic of any character destroying the lower animals, and soon heaven and earth are moved to suppress it. See the tender solicitude, the perverted sympathy that is at once manifested in them; and yet the fact that one seventh of our population should be victims of so insidious a foe is accepted with the utmost complacency and indifference! The church has its missionaries, medicine should have hers. Men are needed, "great men and true," who will preach the curability and preventability of consumption, and strive to impress upon their listeners the necessity of constructing and maintaining sanitary houses that its devastation may be checked. The public having awakened to a realization of the importance of these measures, legislators will then yield to the pressure brought to bear upon them and stand ready to advocate and enact the laws demanded by their constituents.

Self-protection is the only basis on which State charities can be justified. Protection against injury to life and property, or against the loss to society which naturally accrues from enforced idleness or the lack of education, affords an admirable excuse in the dependent insane, deaf, dumb or blind. The danger to health and life from the prevalence of infectious diseases warrants the building of hospitals for their isolation and the lavish expenditure of enormous sums of money for the establishment of quarantine stations. But, as one writer says, "The foundation and support of hospitals simply for the relief of suffering and healing of diseases is, from the standpoint of strict political economy and true statesmanship, a flagrant misuse of public funds, and leads to serious abuses." Every institution of this character should be maintained solely by the community to whose poor it ministers, unless, of course, it be desired to "create a fund for the manufacture of paupers."

It is only within recent years, since the infectiousness of tuberculosis has become an established fact, that the sanitarian is in a position to petition the State to provide for its indigent consumptives. When it eventuated from heredity and environment the duty to the individual presented another and a different aspect. If smallpox, diphtheria or scarlet fever prevailed the community became at once aroused and eradicable measures were resorted to; but little if any attention was paid the pitiable victims of the white plague. Man had to die, and why not from consumption as anything else? He could not select the method and, consequently, should accept the inevitable with resignation. But the times and thought have changed, and with them, fortunately, our conceptions of tuberculosis. Confident of our position, we proclaim without fear of refutation that it is infectious, and therefore, communicable. The situation presents another phase; and the State, the protector of the physical well-being and advancement of its citizens, becomes at once, as the champion of preventive medicine, the logical and moral custodian of their health. It consequently becomes almost imperative that an appropriation should be set aside to blot out from our midst this the most potent enemy of mankind. Sanatoria are a necessity, and special institutions to treat the curable and isolate the hopeless consumptive poor indispensable.

Construct model houses, on good soil, with good drainage and hygienic surroundings, where crowding is impossible and privacy assured; give to the workingman a cleaner, healthier, pleasanter, more comfortable house, and the rumshop—not infrequently responsible for many premature deaths—will have fewer devotees.

In speaking of the right of the State to provide hospitals for its tuberculous patients, a celebrated barrister voices this sentiment: "The paramount object of governments is the protection of their citizens in life, limb, property, and the pursuit of happiness; for this alone the individual pays tribute and renders fealty, and since a diseased person cannot have the perfect enjoyment of either, it is manifest that not only does the State possess the inherent right to secure and preserve the health of its inhabitants, but that such is, perhaps, the highest duty it owes them."

Whatever is in its inception, or afterwards becomes, a public nuisance or a menace to the community may be legally curtailed or abated. The fact that it has existed for ages does not abridge the remedy. The power of the Commonwealth to establish sanatoria and to promulgate and enforce sanitary measures, both directly and indirectly, has been recognized by legislators and authorized by the judiciary. That such is considered one of the most important and vital functions of government is proven by the arbitrary control vested by the people in public boards of health, who regulate the movements of citizens under given conditions, superintend both the importation and exportation of merchandise, and summarily condemn or destroy private property. This power is limited only by the exigency of the situation and the courts, if appealed to, will sanction and applaud their acts. The duty of the State, therefore, comprehends the adoption of all means known to science for the cure, control and eradication of dangerous diseases, while its authority ends only by legislative discretion or is influenced by the resources of the community.

The poor are as deserving as the rich. One God has made us all; and in this land of ours "all men are born free and equal." Some have been given wealth and the means necessary to command every luxury that opulence can afford; others, in their pitiable poverty, live their lives of sorrow and care and approach a premature grave uncared for, unhonored and unsung, while time, unmindful of the magnitude of their afflictions and of the countless thousands doomed to follow in their wake, speeds on without shedding a tear of sympathy. For these latter we plead especially. To them the State owes a debt that is as genuine and urgent as any that may be due its other unfortunates. Moreover, by saving the lives of a portion of these patients, and circumscribing the spread of tuberculosis, the Commonwealth will, in the end, win for itself a merited honor, and many lives be spared to add to the glory of its immortality. Besides, " personal protection is the fundamental idea of unselfish charity, "and this, I take it, is the principal object of this organization.

